# An Empirical Likelihood Method for Semiparametric Linear Regression with Right Censored Data

**DOI:** 10.1155/2013/469373

**Published:** 2013-03-14

**Authors:** Kai-Tai Fang, Gang Li, Xuyang Lu, Hong Qin

**Affiliations:** ^1^Beijing Normal University-Hong Kong Baptist University, United International College, Zhuhai 519085, China; ^2^Department of Biostatistics, School of Public Health, University of California, Los Angeles, CA 90095-1772, USA; ^3^Department of Statistics, Central China Normal University, Wuhan 430079, China

## Abstract

This paper develops a new empirical likelihood method for semiparametric linear regression with a completely unknown error distribution and right censored survival data. The method is based on the Buckley-James (1979) estimating equation. It inherits some appealing properties of the complete data empirical likelihood method. For example, it does not require variance estimation which is problematic for the Buckley-James estimator. We also extend our method to incorporate auxiliary information. We compare our method with the synthetic data empirical likelihood of Li and Wang (2003) using simulations. We also illustrate our method using Stanford heart transplantation data.

## 1. Introduction

Suppose that one observes right censored regression data consisting of *n* i.i.d. triples (*X*_*i*_, *Z*_*i*_, *δ*_*i*_) = (*X*_*i*_, *Y*_*i*_∧*C*_*i*_, *I*[*Y*_*i*_ ≤ *C*_*i*_]), *i* = 1,…, *n*, where for subject *i*, *Y*_*i*_ is a known monotone transformation of the survival time of interest, *C*_*i*_ is the corresponding censoring time, and *X*_*i*_ = (*X*_*i*1_,…, *X*_*ip*_)^*τ*^ is a vector of *p* covariates. We consider the problem of making inferences for the slope parameter of the semiparametric linear regression model as follows:

(1) $$\begin{array}{c} Y_{i}=X_{i}^{\tau }\beta +\epsilon_{i},\;i=1,\ldots ,n, \end{array}$$

where *β* = (*β*_1_,…, *β*_*p*_)^*τ*^ is a *p* × 1 vector of unknown regression coefficients and *ϵ*_*i*_'s are independent and identically distributed random errors with an unknown distribution *F*. Assume that *ϵ*_*i*_ is independent of (*X*_*i*_, *C*_*i*_) and that conditional on *X*_*i*_, *Y*_*i*_ and *C*_*i*_ are independent, *i* = 1,…, *n*. Because the error distribution *F* is completely unknown, we assume no intercept term in model (1) in order for *F* to be identifiable. Assume further that the covariance matrix of *X*_*i*_ is positive definite. Model (1) provides a useful alternative to the popular Cox [1] model when the proportional hazards assumption does not hold. Furthermore, it makes no parametric assumption on the error distribution and is, thus, more flexible than parametric accelerated failure time models (cf. Andersen et al. [2]).

Buckley and James [3] extended the least squares method to estimate the regression coefficient *β* in model (1) with right censored data. The Buckley-James estimator can be calculated using an iterative algorithm. Its consistency and asymptotic normality have been established by Lai and Ying [4], Ritov [5], Tsiatis [6], Ying [7], and others. However, its asymptotic variance involves the unknown hazard function of *ϵ*, and its derivatives whose nonparametric estimations are problematic (cf. Ritov [5]). To overcome this problem, several approaches have been studied in the literature. Jin et al. [3, 8], Wei et al. [9], and Lin and Geyer [10] considered rank-based inferences. Koul et al. [11] introduced a synthetic data approach which was further investigated by Zhou [12], Lai et al. [13], and others. Let *Y*_*iG*_ = *δ*_*i*_ *Y*_*i*_/*G*(*Y*_*i*_−), where *G* is the survival function of the censoring time. It can be shown that *E*(*Y*_*iG*_ | *X*_*i*_) = *E*(*Y*_*i*_ | *X*_*i*_) if *C* is independent of *X* and *Y*. Koul et al. [11] proposed to estimate the regression parameters by regressing $Y_{i\hat{G}}$ on *X*_*i*_, where $\hat{G}$ is the Kaplan-Meier [14] estimate of *G* and $Y_{i\hat{G}}$ is referred to as a synthetic variable. Koul et al. [11] showed that the synthetic data estimator is asymptotically normal and its variance is simple to estimate. However, it requires a strong assumption that the censoring time is independent of both the survival time and the covariates. It can also have poor small sample performance in the presence of heavy censoring (cf. Li and Wang [15]). Jing and Qin [16] and Li and Wang [15] independently developed empirical likelihood-based inference for *β* using synthetic data. Their methods provide substantial improvement over the normal theory method of Koul et al. [11], but are still not very satisfactory for small samples (cf. Li and Wang [15]). The synthetic data approach makes an independence assumption between *C* and (*Y*, *X*). Li and Lu [17] showed that it can yield seriously biased parameter estimate if *C* depends on *X*. Zhou and Li [18] developed a censored data EL method under a weaker censorship assumption that *Y* and *C* are conditionally independent given *X*. Their method also demonstrated better small sample performance than the synthetic data EL method when the errors *ϵ*_*i*_'s are independent and identically distributed (homogeneous). On the other hand, it is not easy to extend the method of Zhou and Li [18] to incorporate auxiliary information and to construct confidence regions for linear combinations of the regression coefficients, which are relatively easy using the synthetic data EL method (cf. Li and Wang [15]).

In this paper, we develop a new empirical likelihood method for linear regression with right censored data. We construct an estimated empirical likelihood based on the estimation equation for the Buckley-James [3] estimator. The approach inherits many appealing features of empirical likelihood. For example, the shape and orientation of a confidence region are entirely determined by data. Most importantly, it does not involve variance estimation. Our simulation shows that, under the assumptions of model (1), our method is far superior to the synthetic data empirical likelihood method of Li and Wang [15], with substantially shorter confidence intervals and higher coverage probabilities. Compared to Zhou and Li [18]'s method, our method may not be as efficient, but it is easier to compute. It can also be extended easily to incorporate auxiliary information and to construct confidence regions for linear combinations of the regression coefficients. More discussion of the pros and cons of our method in relation to existing methods are given later in Remark 2.

The use of empirical likelihood dates back at least to Thomas and Grunkemeier [19] who constructed confidence intervals for survival probabilities. The idea was later popularized by Owen [20, 21] who derived confidence regions for the mean of a random vector. Since the work of Owen [20, 21], there has been extensive developments of empirical likelihood methods for a wide range of applications including, among others, linear models [22–24], generalized linear models [25], quantile estimation [26], biased sample models [27], generalized estimating equations [28], dependent process model [29], partial linear models [30], mixture proportions [31], confidence bands for survival functions [32], confidence bands for quantile functions [33], censored cost regression [34], and confidence tubes for multiple quantile plots [35]. Some nice discussion of properties of empirical likelihood can be found in DiCiccio, Hall and Romano [36], Hall [37], and Hall and Scala [38], and others. A comprehensive survey of empirical likelihood and further references can be found in Owen [39] and Li et al. [40].

In Section 2, we derive an estimated empirical likelihood for *β* by combining the ideas of Owen [21, 22] and Buckley and James [3]. An adjustment factor is adopted so that the adjusted empirical likelihood has an asymptotic standard Chi-square distribution. We also discuss how to incorporate auxiliary information using empirical likelihood.  Section 3 presents results from a simulation study to illustrate the performance of our method compared with the synthetic data empirical likelihood method. A real data example is also provided.  Section 4 gives some concluding remarks. The proofs are collected in the appendix.

## 2. Empirical Likelihood Inference

### 2.1. Empirical Likelihood Based on the Buckley-James Estimating Equation

We motivate our procedure by first considering the case where *μ*_*X*_ = *E*(*X*) and *F* are known.

We first give a review of the Buckley-James equation. It can be shown that, under model (1),

(2) $$\begin{array}{c} \beta ={\left[E\left\{\left(X_{i}-\mu_{X}\right)X_{i}\right\}\right]}^{-1}E\left\{\left(X_{i}-\mu_{X}\right)Y_{i}\right\}, \end{array}$$

or, equivalently,

(3) $$\begin{array}{c} E\left\{\left(X_{i}-\mu_{X}\right)\left(Y_{i}-X_{i}^{\tau }\beta \right)\right\}=0. \end{array}$$

Because *Y*_*i*_ is not always observable, we impute *Y*_*i*_ by its conditional expectation given the observed data as follows:

(4) Yi∗=E(Yi ∣ Xi,Zi,δi)=δiZi+(1−δi) ×{Xiτβ+∫Zi−Xiτβ∞tdF(t)1−F(Zi−Xiτβ)}.

Noting that *E*(*Y*_i_* | *X*_*i*_) = *E*(*Y*_*i*_ | *X*_*i*_), we can replace *Y*_*i*_ by *Y*_i_* in (3). This leads to

(5) $$\begin{array}{c} E\left\{W_{i}\left(\beta ,F,\mu_{X}\right)\right\}=0, \end{array}$$

where

(6) Wi(β,F,μX)=(Xi−μX)×{δi(Zi−Xiτβ)  +(1−δi)∫Zi−Xiτβ∞tdF(t)1−F(Zi−Xiτβ)}.

Now, the problem of testing *H*_0_ : *β* = *β*_0_ is equivalent to testing

(7) $$\begin{array}{c} H_{0}:E\left\{W_{i}\left(\beta_{0},F,\mu_{X}\right)\right\}=0, \end{array}$$

based on *n* i.i.d. observations *W*_*i*_(*β*_0_, *F*, *μ*_*X*_), *i* = 1,…, *n*. This problem can be readily solved using the results of Owen [21]. Specifically, define

(8) ln(β,F,μX)=−2sup⁡{∑i=1nlog⁡(npi) ∣ ∑i=1npiWi(β,F,μX)=0,        ∑i=1npi=1,  pi≥0,  1≤i≤n}.

Owen [21] showed that

(9) $$\begin{array}{c} l_{n}\left(\beta ,F,\mu_{X}\right)=2\sum_{i=1}^{n}\log\left\{1+\lambda^{\tau }W_{i}\left(\beta ,F,\mu_{X}\right)\right\}, \end{array}$$

where *λ* is the solution of the equation

(10) $$\begin{array}{c} \frac{1}{n}\sum_{i=1}^{n}\;\frac{W_{i}\left(\beta ,F,\mu_{X}\right)}{1+\lambda^{\tau }W_{i}\left(\beta ,F,\mu_{X}\right)}=0. \end{array}$$

Moreover, under *H*_0_, *l*_*n*_(*β*_0_, *F*, *μ*_*X*_) has an asymptotic central Chi-square distribution with *p* degrees of freedom. Thus, one would reject *H*_0_ if *l*_*n*_(*β*_0_, *F*, *μ*_*X*_) > *χ*_*p*,*α*_^2^, where *χ*_*p*,*α*_^2^ is the upper *α* quantile of the standard Chi-square distribution with *p* degrees of freedom.

Now, we consider the case where both *F* and *μ*_*X*_ are unknown. Define *e*_*i*_^*β*^ = *Z*_*i*_ − *X*_*i*_^*τ*^ *β*, *i* = 1,…, *n*. We estimate *F* by

(11) $$\begin{array}{c} {\hat{F}}_{n}^{\beta }\left(t\right)=1-\prod_{i=1}^{n}{\left[\frac{n-i}{n-i+1}\right]}^{I[e_{\left(i\right)}^{\beta }\leq t,\delta_{\left(i\right)}=1]}, \end{array}$$

the Kaplan-Meier [14] estimator of *F* based on {(*e*_*i*_^*β*^, *δ*_*i*_), *i* = 1,…, *n*}, where *e*_(1)_^*β*^ ≤ ⋯≤*e*_(*n*)_^*β*^ are the order statistics of the *e* ^*β*^-sample, and *δ*_(*i*)_ is the *δ* associated with *e*_(*i*)_^*β*^, *i* = 1,…, *n*. In addition, we estimate *μ*_*X*_ by the sample mean $\overset{-}{X}=n^{-1}\sum_{i=1}^{n}X_{i}$.

We propose to use $l_{n}(\beta_{0},{\hat{F}}_{n}^{\beta_{0}},\overset{-}{X})$ as a likelihood ratio statistic for testing *H*_0_. However, we can no longer use Owen's [21] result for the null limiting distribution of $l_{n}(\beta_{0},{\hat{F}}_{n}^{\beta_{0}},\overset{-}{X})$ because $W_{i}(\beta ,{\hat{F}}_{n}^{\beta },\overset{-}{X})$'s are not i.i.d. The following theorem states that an adjustment factor is needed so that the limiting null distribution is standard Chi-squared.

**Theorem**

Assume that the conditions listed in the appendix hold. Define that

(12) $$\begin{array}{c} c_{n}\left(\beta \right)=\frac{\mathrm{tr}\left({\hat{\Sigma }}_{2}^{-1}\left(\beta \right)S_{n}\left(\beta \right)\right)}{\mathrm{tr}\left({\hat{\Sigma }}_{1}^{-1}\left(\beta \right)S_{n}\left(\beta \right)\right)}, \end{array}$$

where

(13) Sn(β)={∑i=1nWi(β,F^nβ,X−)}{∑i=1nWi(β,F^nβ,X−)}τ,Σ^1(β)=1n∑i=1nWi(β,F^nβ,X−)Wi(β,F^nβ,X−)τ,Σ^2(β)=1n∑i=1n∫0∞(u−∫u∞vdFnβ(v)1−Fnβ(u))2   ×V^(u)dNi(u),V^(u)=∑i=1n{Xi−X−(u)}{Xi−X−(u)}τYi(u)∑j=1nYj(u),X−(u)=∑i=1nXiYi(u)∑j=1nYj(u),Ni(u)=I(eiβ≤u,δi=1),Yi(u)=I(eiβ≥u).

Then, under *H*_0_ : *β* = *β*_0_,

(14) $$\begin{array}{c} c_{n}\left(\beta_{0}\right)l_{n}\left(\beta_{0},{\hat{F}}_{n}^{\beta_{0}},\overset{-}{X}\right)\overset{d}{\to }\chi_{p}^{2}, \end{array}$$

where *χ*_*p*_^2^ is a standard Chi-square random variable with *p* degrees of freedom.

It follows immediately that an approximate *α*-level test rejects *H*_0_ if

(15) $$\begin{array}{c} c_{n}\left(\beta_{0}\right)l_{n}\left(\beta_{0},{\hat{F}}_{n}^{\beta_{0}},\overset{-}{X}\right)>\chi_{p,\alpha }^{2}. \end{array}$$

Moreover, an approximate 1 − *α* confidence region for *β* is given by

(16) $$\begin{array}{c} \left\{\beta :c_{n}\left(\beta \right)l_{n}\left(\beta ,{\hat{F}}_{n}^{\beta },\overset{-}{X}\right)\left(\beta \right)\leq \chi_{p,\alpha }^{2}\right\}. \end{array}$$

**Remark 2**

Although both the above derived method and Zhou and Li [18] use the Buckley-James estimation equation, a sample version of (7), they are different in that we use the complete data likelihood, whereas Zhou and Li [18] uses the exact censored data likelihood to construct the EL. Similar to Li and Wang [15], the above method can be extended to incorporate auxiliary information and to obtain EL procedure for a subset, contrast, or linear combinations of the regression coefficients, which does not seem easy when using the method of Zhou and Li [18]. As an illustration, we show below how to extend our method to incorporate auxiliary information.

### 2.2. Empirical Likelihood with Auxiliary Information

 Auxiliary population characteristics of the covariate *X* are sometimes available in practice. Effective usage of the auxiliary information can lead to more efficient inference (cf. Chen and Qin [41], Qin and Lawless [28] and Zhang [42, 43]). Here, we show how to use empirical likelihood to incorporate auxiliary information of *X*.

Assume that the available auxiliary information on *X* is given in the form *Eg*(*X*) = 0, where *g*(*x*) = (*g*_1_(*x*),…, *g*_*r*_(*x*))^*τ*^, *r* ≥ 1, is a vector of *r* known functions. To make use of the auxiliary information, we maximize

(17) $$\begin{array}{c} \prod_{i=1}^{n}p_{i} \end{array}$$

subject to ∑_*i*=1_^*n*^ *p*_*i*_ = 1, ∑_*i*=1_^*n*^ *p*_*i*_ *g*(*X*_*i*_) = 0, and $\sum_{i=1}^{n}p_{i}W_{i}(\beta ,{\hat{F}}_{n}^{\beta },\overset{-}{X})=0$.

Let $A_{ni}(\beta )={\left(g^{\tau }(X_{i}),W_{i}{\left(\beta ,{\hat{F}}_{n}^{\beta },\overset{-}{X}\right)}^{\tau }\right)}^{\tau }$. By the method of Lagrange multipliers, it can be shown that (17) is maximized at

(18) $$\begin{array}{c} p_{in}=\frac{1}{n}\frac{A_{ni}\left(\beta \right)}{1+\zeta_{n}^{\tau }A_{ni}\left(\beta \right)},\;\;\;i=1,\ldots ,n, \end{array}$$

where *ζ*_*n*_ satisfies the following equation

(19) $$\begin{array}{c} \frac{1}{n}\sum_{i=1}^{n}\frac{A_{ni}\left(\beta \right)}{1+\zeta_{n}^{\tau }A_{ni}\left(\beta \right)}=0. \end{array}$$

Hence, the empirical log-likelihood ratio function for *β* is given by

(20) $$\begin{array}{c} l_{n,AU}\left(\beta \right)=-2\sum_{i=1}^{n}\log\;np_{in}=2\sum_{i=1}^{n}\log\left(1+\zeta_{n}^{\tau }A_{ni}\left(\beta \right)\right). \end{array}$$

Similar to the previous section, an adjustment factor is needed for *l*_*n*,*AU*_(*β*) to have a standard Chi-square asymptotic distribution, as stated in the following theorem.

**Theorem**

Assume that *V*_1_ = *Eg*(*X*_*i*_)*g* ^*τ*^(*X*_*i*_) is positive definite and that *Eg*(*X*)*W*_*i*_(*β*, *F*, *μ*_*X*_)^*τ*^) exists. Define that *V*_*n*1_(*β*) = (1/*n*)∑_*i*=1_^*n*^ *g*(*X*_*i*_)*g* ^*τ*^(*X*_*i*_),

(21) Vn2(β)=1n∑i=1ng(Xi)Wi(β,F^nβ,X−),Vn1,AU(β)=(Vn1(β),Vn2(β)Vn2τ(β),Σ^1(β)),Vn2,AU(β)=(Vn1(β),00Σ^2(β)),

where ${\hat{\Sigma }}_{1}(\beta )$ and ${\hat{\Sigma }}_{2}(\beta )$ are defined in Theorem 1. Then, under the conditions of Theorem 1,

(22) $$\begin{array}{c} c_{n,AU}\left(\beta_{0}\right)l_{n,AU}\left(\beta_{0}\right)\overset{d}{\to }\chi_{p+r}^{2}, \end{array}$$

as *n* → *∞*, where

(23) $$\begin{array}{c} c_{n,AU}\left(\beta \right)=\frac{\mathrm{tr}\left(V_{n2,AU}^{-1}\left(\beta \right)\Psi_{n}\left(\beta \right)\right)}{\mathrm{tr}\left(V_{n1,AU}^{-1}\left(\beta \right)\Psi_{n}\left(\beta \right)\right)}\; \end{array}$$

and Ψ_*n*_(*β*) = (∑_*i*=1_^*n*^ *A*_*ni*_(*β*))(∑_*i*=1_^*n*^ *A*_*ni*_(*β*))^*τ*^.

## 3. Numerical Results

We carried out Monte Carlo simulations to examine the performance of our proposed empirical likelihood method based on the Buckley-James estimating equation (ELEE) in comparison to the empirical likelihood method based on synthetic data (ELSD) [15, 16]. We considered five models. In model A, the data were generated from *Y* = 1 + *X* + *ϵ*, where *X* and *ϵ* are independent normal random variables with mean 0 and variances 0.25, respectively, the censoring time *C* is a normal random variable with mean *μ* and standard deviation 4. Model B is the same as model A except that *X*~ Bernoulli(0.5) − 0.5. Model C assumes that *Y* = *X* + *ϵ*, where *X* ~ *N*(0,0.5^2^), *ϵ* ~ Weibull (shape = 1.843, scale = 1), and *C* ~ *N*(*μ*, 4^2^). Model D is the same as model A except that *C* ~ *N*(*μ* + 2*X*, 15), allowing the censoring time to depend on *X*. Model E is the same as model A except that *ϵ* ~ *N*(0, *X* ^2^), allowing for heterogeneous errors. We adjust *μ* to produce different censoring rate (CR). We also vary the sample size *n*. The achieved confidence levels and average lengths of the ELEE and ELSD confidence intervals for the slope parameter are summarized in Table 1. Each entry in the table was computed using 3,000 Monte Carlo samples.

We see from Table 1 that under models A–D, the coverage probabilities of ELEE method are consistently close to or slightly above the nominal level, whereas the ELSD method can have severe under-coverage for small samples (*n* = 50) and large censoring rate (75%). Furthermore, the ELEE confidence intervals are much narrower than the ELSD confidence intervals. In particular, the ELSD method failed completely with unreasonably low coverage under model D when the censoring time is dependent on *X*. On the other hand, under model E with heterogeneous errors and independent censoring time, ELEE showed larger coverage probability errors than ELSD, as one would have expected. Thus, the ELEE method seems to dominate the ELSD method when the errors are homogeneous, but can be outperformed by ELSD in the presence of heterogeneous errors.

We now illustrate our method using the Stanford heart transplant data (Miller [44], Table 1). The data include the lengths of survival (in days) after transplantation, ages at time of transplant, and T5 mismatch scores for 69 patients who received heart transplants at Stanford and were followed to April 1, 1974. Twenty-four patients were still alive on April 1, 1974 and thus their survival times were censored. For illustration purpose, we considered two models, labeled as (I) and (II), respectively, where the dependent variable *Y* is the logarithm to base 10 of the length of survival from transplantation. Specifically, model (I) regresses *Y* on the mismatch score T5, and model (II) regresses *Y* on age. As in Koul et al. [11], regression of survival on the mismatch score T5 was performed with nonrejection-related death being treated as censoring since the mismatch score is directed at the rejection phenomenon [44].  Table 2 reports the parameter estimates and 95% confidence intervals for the slope parameters using our empirical likelihood method based on the Buckley-James estimating equation (ELEE) and the empirical likelihood method based on synthetic data (ELSD) [15, 16].

It is seen from Table 2 that both the point estimates and confidence intervals are quite different between the ELEE and ELSD methods for this data. For example, the KSV slope estimates of T5 and age are positive, which seems to contradict to the common belief that the survival time tends to be negatively correlated with T5 (the mismatch score) and age at diagnosis. For model (II), the ELSD confidence interval does not include zero and thus concludes a significant age effect at the 5% significant level, whereas the ELEE method does not produce a significant result. The ELSD results could be misleading in this example since the censoring time appears to depend on age and T5 as pointed out by Leurgans [45] and Li and Lu [17]. We have also examined the Cox-Snell residual plots for a number of parametric accelerated failure time (AFT) models. We found that the log-normal AFT model fits the data fairly well which indicates that the semiparametric AFT model should also fit the data well.

## 4. Discussion

 This research adds a new tool to the toolbox of empirical likelihood (EL) methods for linear regression with right censored data. The three EL methods, namely, the method of Li and Wang [15], the method of Zhou and Li [18], and the method of this paper should be regarded as complementary, as opposed to competing, methods for linear regression with right censored survival data. Each of the three approaches has its own merits and shortcomings. None dominates the others in every aspect. So it is important to understand the pros and cons of these methods. First of all, if the errors in the regression model are i.i.d (homogeneous) and the censoring time is conditionally independent of the survival time given the covariates, then the method of Li and Zhou [18] and the method of this paper are expected to be superior to the Li and Wang [15] method, as demonstrated by simulations in Table 1 and Li and Zhou [18]. This is because the former two methods use the Buckley-James estimating equations which take advantage of the homogeneous error assumption to implicitly impute a censored observation from the model using all residuals, whereas the latter uses synthetic data that does not utilize the homogeneous error assumption. Furthermore, based on our limited experience, the Li and Zhou [18] seems to be more efficient than the method of this paper. This is not a surprise since, Li and Zhou method [18] uses the exact censored likelihood, whereas the ELEE method of this paper uses an approximate likelihood. On the other hand, Li and Zhou [18] only developed an EL procedure for the whole vector of regression coefficients and did not discuss how to extend their method to incorporate auxiliary information which can be easily done using the approach of this paper as described in Section 2.2. Secondly, if the errors are heterogeneous, then the Li and Zhou [18] method and the method of this paper are expected to fail as indicated by Table 1 (model E), but the synthetic data method of Li and Wang [15] would still be asymptotically valid under the stronger censorship assumption that the censoring time is independent of both the survival time and the covariates. Finally, the method of Li and Wang [15] can fail completely when the censoring time is dependent on the covariate as shown in Table 1 (model D).

Some further extensions are warranted in future research. For example, the method of this paper can be extended to draw inference for linear combinations of the regression coefficients along the lines of Li and Wang ([15, Section 3]). It can also be extended to construct EL confidence regions based on estimating equations for a class of M-estimators described by Ritov [5]. Finally, this paper focuses only on EL methods for right censored data. It would be interesting to investigate how these EL methods compare to other methods such as the rank-based regression methods in future studies.

## Figures and Tables

**Table 1:** Comparison of the coverage probability (CP) and average width (Width) of two empirical likelihood confidence intervals for the slope parameter under four different models with various sample size (*n*) and censoring rate (CR). Here, ELEE is the proposed method, and ELSD is the method of Li and Wang [15]. Each entry is based on 3,000 Monte Carol samples.

			Nominal level = 90%				Nominal level = 95%			
Model	*n*	CR	CP		width		CP		Width	
			ELEE	ELSD	ELEE	ELSD	ELEE	ELSD	ELEE	ELSD
	50	0.75	0.94	0.77	1.59	2.18	0.98	0.84	1.95	2.70
	100	0.75	0.94	0.82	0.85	1.66	0.97	0.89	1.03	2.02
	500	0.75	0.91	0.88	0.31	0.81	0.96	0.94	0.37	0.97
	50	0.3	0.94	0.87	0.69	1.30	0.97	0.92	0.82	1.55
A	100	0.3	0.93	0.89	0.45	0.94	0.97	0.94	0.53	1.12
	500	0.3	0.91	0.90	0.18	0.43	0.96	0.95	0.21	0.51
	50	0.1	0.95	0.88	0.59	1.10	0.98	0.93	0.70	1.30
	100	0.1	0.93	0.89	0.39	0.79	0.97	0.94	0.47	0.94
	500	0.1	0.90	0.90	0.16	0.36	0.95	0.95	0.19	0.43

	50	0.75	0.93	0.83	1.20	2.05	0.95	0.88	1.40	2.50
	100	0.75	0.94	0.87	0.77	1.49	0.97	0.92	0.92	1.80
	500	0.75	0.93	0.89	0.30	0.67	0.96	0.94	0.36	0.80
	50	0.3	0.95	0.88	0.66	1.23	0.98	0.94	0.78	1.48
B	100	0.3	0.94	0.90	0.44	0.88	0.97	0.95	0.52	1.05
	500	0.3	0.92	0.90	0.18	0.39	0.96	0.95	0.21	0.47
	50	0.1	0.94	0.89	0.58	1.08	0.97	0.95	0.69	1.29
	100	0.1	0.94	0.90	0.39	0.77	0.97	0.94	0.46	0.92
	500	0.1	0.91	0.91	0.16	0.35	0.96	0.95	0.19	0.41

	50	0.75	0.93	0.77	1.49	2.01	0.96	0.83	2.01	2.46
	100	0.75	0.93	0.82	0.80	1.56	0.97	0.88	0.97	1.90
	500	0.75	0.92	0.87	0.29	0.76	0.96	0.93	0.35	0.92
	50	0.3	0.93	0.86	0.67	1.21	0.97	0.92	0.81	1.45
C	100	0.3	0.93	0.88	0.44	0.88	0.97	0.93	0.53	1.05
	500	0.3	0.91	0.89	0.18	0.40	0.96	0.94	0.21	0.48
	50	0.1	0.94	0.87	0.60	1.01	0.97	0.93	0.71	1.21
	100	0.1	0.93	0.89	0.39	0.73	0.97	0.94	0.47	0.87
	500	0.1	0.92	0.90	0.16	0.33	0.96	0.95	0.19	0.39

	50	0.75	0.95	0.68	1.74	2.45	0.97	0.77	1.87	2.98
	100	0.75	0.94	0.60	0.83	1.83	0.97	0.69	1.01	2.21
	500	0.75	0.92	0.12	0.30	0.89	0.96	0.18	0.36	1.06
	50	0.3	0.94	0.81	0.68	1.31	0.97	0.88	0.82	1.56
D	100	0.3	0.93	0.76	0.45	0.94	0.97	0.84	0.53	1.12
	500	0.3	0.91	0.39	0.18	0.43	0.96	0.51	0.21	0.51
	50	0.1	0.94	0.86	0.59	1.09	0.97	0.92	0.71	1.30
	100	0.1	0.94	0.87	0.39	0.78	0.97	0.93	0.47	0.93
	500	0.1	0.91	0.78	0.16	0.36	0.95	0.86	0.19	0.42

	50	0.75	0.78	0.76	1.52	2.25	0.85	0.83	1.79	2.75
	100	0.75	0.78	0.80	0.85	1.74	0.85	0.87	0.62	2.11
	500	0.75	0.73	0.85	0.34	0.88	0.81	0.92	0.40	1.06
	50	0.3	0.81	0.85	0.76	1.43	0.87	0.91	0.89	1.71
E	100	0.3	0.79	0.87	0.53	1.05	0.86	0.92	0.62	1.26
	500	0.3	0.77	0.89	0.22	0.50	0.84	0.94	0.26	0.60
	50	0.1	0.81	0.86	0.69	1.24	0.87	0.92	0.81	1.48
	100	0.1	0.80	0.87	0.49	0.91	0.86	0.93	0.57	1.08
	500	0.1	0.76	0.89	0.20	0.42	0.83	0.94	0.23	0.50

Model A: *Y* = 1 + *X* + *ϵ*, where *X* ~ *N*(0,0.5^2^), *ϵ* ~ *N*(0,0.5^2^), and *C* ~ *N*(*μ*, 4^2^); model B: *Y* = 1 + *X* + *ϵ*, where *X*∼ Bernoulli(0.5) − 0.5, *ϵ* ~ *N*(0,0.5^2^), and *C* ~ *N*(*μ*, 4^2^); model C: *Y* = *X* + *ϵ*, where *X* ~ *N*(0,0.5^2^), *ϵ*∼ Weibull (shape = 1.843, scale = 1), and *C* ~ *N*(*μ*, 4^2^); model D (Dependent censoring): *Y* = 1 + *X* + *ϵ*, where *X* ~ *N*(0,0.5^2^), *ϵ* ~ *N*(0,0.5^2^), and *C* ~ *N*(*μ* + 2*X*, 15); model E: *Y* = 1 + *X* + *ϵ*, where *X* ~ *N*(0,0.5^2^), *ϵ* ~ *N*(0, *X* ^2^), and *C* ~ *N*(*μ*, 4^2^).

**Table 2:** Empirical likelihood confidence interval estimates for heart transplant data. Nominal level = 95%.

Model		Parameter estimates		Confidence intervals	
	Parameter	BJ	KSV	ELEE	ELSD
(I)	*β*_*T*5_	−0.593	0.258	(−2.740, 0.645)	(−0.596, 0.928)
(II)	*β*_age_	−0.028	0.055	(−0.065, 0.032)	(0.001, 0.128)

Note: BJ refers to the Buckley-James [3] estimate, and KSV refers to the synthetic data estimate of Koul et al. [11]. ELEE is the proposed empirical likelihood method based on the Buckley-James estimating equation, and ELSD is the empirical likelihood method based on synthetic data [15, 16].

## Appendix

Assumption A.1 The covariate *X*_*i*_ has compact support.

Assumption A.2 There exists a function *V*(*u*) such that $\hat{V}(u)$ converges uniformly to *V*(*u*) in probability as *n* → *∞*.

The following lemmas are needed to prove Theorem 1.

Lemma Let *λ*(*t*) denote the hazard function of *F*. Then,

(A.1) $$\begin{array}{c} n^{-1/2}\sum_{i=1}^{n}W_{i}\left(\beta ,{\hat{F}}_{n}^{\beta },\overset{-}{X}\right)\overset{ℒ}{\to }N\left(0,\Sigma_{2}\left(\beta \right)\right), \end{array}$$

where Σ_2_(*β*) = ∫_0_^*∞*^{*w*(*t*)}^2^ *V*(*t*)*λ*(*t*)*dt* and

(A.2) $$\begin{array}{c} w\left(t;F\right)=t-\frac{\int_{t}^{\infty }udF\left(u\right)}{1-F\left(t\right)}. \end{array}$$

Moreover, Σ_2_(*β*) can be consistently estimated by ${\hat{\Sigma }}_{2}(\beta )$.

Proof By Proposition 4.1 of Ritov [5]

(A.3) n−1/2∑i=1nWi(β,F^nβ,X−)=n−1/2∑i=1n∫w(t){Xi−X−(t)}dMi(t)+op(1),

where *M*_*i*_(*t*) = *N*_*i*_(*t*) − ∫_0_^*t*^ *Y*_*i*_(*u*)*λ*(*u*)*du*, *i* = 1,…, *n*, are orthogonal locally square integrable martingales with respect to the filtration *ℱ*_*n*_(*t*) = *σ*[*I*(*e*_*i*_^*β*^ ≤ *t*), *δ*_*i*_ *I*(*e*_*i*_^*β*^ ≤ *t*), *X*_*i*_; *i* = 1,…, *n*]. This, together with Rebolledo's martingale central limit theorem (cf. Andersen et al. [2]), proves (A.1).

Lemma Under the conditions of Theorem 1,

- $\max_{1\leq i\leq n}||W_{i}(\beta ,{\hat{F}}_{n}^{\beta },\overset{-}{X})||=o_{p}(n^{1/2})$,
- *λ* = *O*_*p*_(*n* ^−1/2^).

Proof (a) By Assumption A.1, $||X_{i}-\overset{-}{X}||\leq K$ for some constant *K*. Thus,

(A.4) max⁡1≤i≤n||Wi(β,F^nβ,X−)|| ≤K max⁡1≤i≤n|δiϵiβ|  +K max⁡1≤i≤n|(1−δi){eiβ−w(eiβ;F)}|  +K max⁡1≤i≤n|1−δi||w(eiβ;Fnβ)−w(eiβ;F)| ≤o(n1/2)+o(n1/2)+Ksup⁡t|w(t;Fnβ)−w(t;F)| =o(n1/2)+Op(n−1/2)=o(n1/2),

where we have used Lemma 3 of Owen [21] in the second step and Lemma 4.1 of Ritov [5] in the third step. (b) Define that ${\tilde{\Sigma }}_{1}(\beta )=(1/n)\sum_{i=1}^{n}W_{i}(\beta ,F,\mu_{X})W_{i}{\left(\beta ,F,\mu_{X}\right)}^{\tau }$. Then, applying Lemma 4.1 of Ritov [5], it can be shown that

(A.5) $$\begin{array}{c} {\hat{\Sigma }}_{1}\left(\beta \right)={\tilde{\Sigma }}_{1}\left(\beta \right)+o_{p}\left(1\right). \end{array}$$

Let *λ* = *ρθ*, where *ρ* ≥ 0 and ||*θ*|| = 1. Then, by Owen [21],

(A.6) $$\begin{array}{c} \theta^{\prime }{\tilde{\Sigma }}_{1}\left(\beta \right)\theta \geq \sigma_{p}+o_{p}\left(1\right), \end{array}$$

where *σ*_*p*_ is the smallest eigenvalue of *E*{*W*_*i*_(*β*, *F*, *μ*)*W*_*i*_(*β*, *F*, *μ*)^*τ*^}. Let *e*_*j*_ be the unit vector in the *j*th coordinate direction. By Lemma A.3,

(A.7) $$\begin{array}{c} ||\frac{1}{n}\sum_{j=1}^{p}e_{j}^{\prime }\sum_{i=1}^{n}W_{in}||=O_{p}\left(n^{-1/2}\right). \end{array}$$

The conclusion of (b) then follows from (10), (A.5)–(A.7), and the arguments used in the proof of (2.14) of Owen [21].

Proof of Theorem 1
 For simplicity, we denote $W_{i}(\beta ,{\hat{F}}_{n}^{\beta },\overset{-}{X})$ by *W*_*i*_ throughout the proof. Applying Taylor's expansion to (9), we have

(A.8) $$\begin{array}{c} {\tilde{l}}_{n}\left(\beta \right)=2\sum_{i=1}^{n}\left(\lambda^{\tau }W_{i}-\frac{1}{2}{\left(\lambda^{\tau }W_{i}\right)}^{2}\right)+r_{n}, \end{array}$$

where

(A.9) $$\begin{array}{c} \;|\;r_{n}\;|\;\;\leq C\sum_{i=1}^{n}{\left(\lambda^{\tau }W_{i}\right)}^{3}\;\text{in}\;\text{probability}. \end{array}$$

Note that

(A.10) $$\begin{array}{c} \left|r_{n}\right|\leq C{||\lambda ||}^{3}\underset{1\leq i\leq n}{\max}||W_{i}||\sum_{i=1}^{n}{||W_{i}||}^{2}. \end{array}$$

This, together with (a), (b), and the fact that

(A.11) 1n∑i=1n‍||Wi||2 ≤1n∑i=1n||Wi(β,F,μX)||2  +1n∑i=1n||Wi(β,F^nβ,X−)−Wi(β,F,μX)||2=Op(1),

implies that

(A.12) $$\begin{array}{c} \left|r_{n}\right|=o_{p}\left(1\right). \end{array}$$

Note that

(A.13) 0=1n∑i=1nWi1+λτWi=1n∑i=1nWi[1−λτWi+(λτWi)21+λτWi]=1n∑i=1nWi−(1n∑i=1nWiWiτ)λ +1n∑i=1nWi(λτWi)21+λτWi.

By (10), (A.5), (A.13), and Lemma A.4, we get

(A.14) λ=(∑i=1nWiWiτ)−1∑i=1nWi+op(n−1/2).

Again by (10), we have

(A.15) 0=∑i=1nλτWi1+λτWi=∑i=1n(λτWi)−∑i=1n(λτWi)2 +1n∑i=1n(λτWi)31+λτWi.

Moreover, by Lemma A.4 and (A.11), we have

(A.16) $$\begin{array}{c} \frac{1}{n}\sum_{i=1}^{n}\frac{{\left(\lambda^{\tau }W_{i}\right)}^{3}}{1+\lambda^{\tau }W_{i}}=o_{p}\left(1\right). \end{array}$$

It then follows from (A.15) and (A.16) that

(A.17) $$\begin{array}{c} \sum_{i=1}^{n}\lambda^{\tau }W_{i}=\sum_{i=1}^{n}{\left(\lambda^{\tau }W_{i}\right)}^{2}+o_{p}\left(1\right). \end{array}$$

Combining (A.8), (A.17) and (A.14) yields the following identity

(A.18) cn(β)ln(β,F^nβ,X−) =(1n∑i=1nWi)τΣ^2−1(β)(1n∑i=1nWi)+op(1).

This, together with Lemma A.3, proves Theorem 1.

The following lemma is needed to prove Theorem 3.

Lemma Assume that the conditions of Theorems 1 and 3 hold. Then,

(A.19) $$\begin{array}{c} \frac{1}{\sqrt{n}}\sum_{i=1}^{n}A_{ni}\left(\beta \right)\overset{ℒ}{\to }N\left(0,V_{2,AU}\left(\beta \right)\right), \end{array}$$

where

(A.20) $$\begin{array}{c} V_{2,AU}\left(\beta \right)=\left(\begin{array}{cc} V_{1}\left(\beta \right), & 0\\ 0, & \Sigma_{2}\left(\beta \right) \end{array}\right). \end{array}$$

Proof This result is a direct consequence of Lemma A.3 and the following facts

(A.21) $$\begin{array}{c} \frac{1}{\sqrt{n}}\sum_{i=1}^{n}g\left(X_{i}\right)\overset{ℒ}{\to }N\left(0,V_{1}\left(\beta \right)\right),\\ \text{Cov}\left(\frac{1}{\sqrt{n}}\sum g\left(X_{i}\right),\frac{1}{\sqrt{n}}\sum_{i=1}^{n}W_{i}\left(\beta ,{\hat{F}}_{n}^{\beta },\overset{-}{X}\right)\right)\to 0. \end{array}$$

Proof of Theorem 3
 The theorem can be proved using Lemma A.5 and along the lines of the proof of Theorem 1. We omit the details.
